# Implementation and evaluation of the WHO maternity care model: a convergent parallel mixed-methods study protocol

**DOI:** 10.3389/fgwh.2024.1309886

**Published:** 2024-04-30

**Authors:** Somayeh Abdolalipour, Shamsi Abbasalizadeh, Sakineh Mohammad-Alizadeh-Charandabi, Fatemeh Abbasalizadeh, Shayesteh Jahanfar, Mojgan Mirghafourvand

**Affiliations:** ^1^ Department of Midwifery, Tabriz University of Medical Sciences, Tabriz, Iran; ^2^ Women's Reproductive Health Research Center, Tabriz University of Medical Sciences, Tabriz, Iran; ^3^ Department of Public Health and Community Medicine, Tufts School of Medicine, Boston, MA, United States

**Keywords:** childbirth experience, childbearing, women’s health, midwifery, fear of childbirth

## Abstract

**Background:**

According to the World Health Organization (WHO), intrapartum care is considered a platform for providing respectful, personalized, and women-centered services to women. This study aims to investigate the intrapartum care model proposed by WHO.

**Methods:**

This convergent parallel mixed-methods study will be carried out in qualitative and quantitative phases. In the quantitative phase (a quasi-experimental study), 108 pregnant women admitted to the maternity ward will be randomized to intervention (receiving intrapartum care based on the WHO model) and control group (receiving routine hospital care) before the beginning of the active stage of labor (cervix dilatation equal to 5 cm) and Wijma's delivery fear scale (DFS) will be completed for them and again at 7–8 cm dilatation. The participants of both groups will be followed up for 6 weeks after labor and then they will be invited to a relatively quiet place to complete the Childbirth Experience Questionnaire (CEQ 2.0), the Edinburgh Postpartum Depression Scale (EPDS), the Post-Traumatic Stress Disorder (PTSD) Symptom Scale (PSS-I), the Pregnancy and Childbirth Questionnaire (PCQ), exclusive breastfeeding and a checklist on willingness to give birth to another child. The qualitative phase will employ content analysis to explain mothers' views about the effects of this model especially subjective components of this model on their labor process after 4–6 weeks. The two phase's results will be discussed in combination.

**Discussion:**

The implementation of such care models is expected to prevent mental disorders caused by negative experiences of childbirth, and also, prevent uncontrolled increases in cesarean sections.

**Clinical Trial Registration:**

https://fa.irct.ir/user/trial/68313/view, identifier (IRCT20120718010324N69).

## Introduction

Pregnancy and childbirth are among the most important experiences of women throughout their lives. The needs and problems of women during pregnancy, labor, and childbirth and the way they are addressed can substantially influence the whole meaning of the fertility process (1). Since mothers never forget their childbirth experiences, providing proper care for pregnant women can create a positive childbirth experience for them with a sense of control, independence, satisfaction, and confidence to mothers and also positively influence their desire to have children again in the future (2). The importance of positive childbirth experiences requires healthcare providers to not only strive to improve the physical health of mothers but also address their mental health (3). A negative childbirth experience can result in post-traumatic stress disorder (PTSD), disordered interpersonal relationships, inefficient mother-infant communication, low rates of exclusive breastfeeding, inappropriate use of maternal and infant care services, fear of future deliveries, and increased desire for elective cesarean section in future pregnancies (4, 5).

Technological advances in obstetrics and gynecology changed childbirth into a biomedical model over the past century (6). Accordingly, traditional labor at home was gradually supplanted by labor at hospitals and medical centers, and childbirth was turned into a medical procedure in most countries that is fully supervised and controlled by a physician (7). However, modern childbirth care has caused pregnant women to consider themselves patients and to lose the independence and control necessary for childbirth management. Midwives can play an important role in providing care that supports the physiologic nature of labor and childbirth, reducing unnecessary interventions and increasing the quality of care for mothers and newborns (8, 9). Therefore, the World Health Organization (WHO) recognized the requirement to introduce appropriate intrapartum care according to evidence-based medicine (10).

Accordingly, WHO in 2015 presented a global perspective in which every pregnant woman and her infant should receive high-quality antenatal, intrapartum, and postpartum care. This initiative also specified how healthcare staff had to provide such services in healthcare systems and how pregnant women and their families had to be treated when receiving such services. In addition, WHO has specified that effective communication, respect, dignity, and emotional support are the main components areas of healthcare quality that should be taken into account for improving the quality of services provided to pregnant women and infants (11).

This organization further emphasized the quality of such services through its 2018 recommendations on intrapartum care for a positive childbirth experience. Going beyond merely the prevention of pregnancy mortality, these recommendations involve a women's rights-based approach to optimizing the health and well-being of pregnant women and their infants (12, 13).

Analysis of the evidence supporting the WHO recommendations on intrapartum care shows that women expect a childbirth experience to meet or go beyond their previous personal and socio-cultural beliefs and expectations (13, 14). According to the recent WHO recommendations, women's experience of intrapartum care is considered an important aspect of high-quality care and not merely a complement to clinical procedures (13). The main principles of the 2018 WHO guidelines that include 56 evidence-based recommendations are as follows: (I) labor and delivery should be personalized and woman-centered, (II) no labor interventions should be done without specific medical indications, (III) only interventions with an immediate purpose that have been proven beneficial should be performed, and (IV) Only one clear goal, which includes the creation of a positive experience for pregnant women as well as their infants and family members, should take precedence over the intrapartum care. 2018 WHO guidelines also provide individual recommendations and how they affect the realization of a positive experience by women (12).

According to the WHO model, intrapartum care is considered a platform for providing respectful, personalized, woman-centered, and effective clinical and non-clinical services to pregnant women and an opportunity for skilled healthcare providers to optimize birth outcomes for women and their infants in a well-functioning system. To achieve these goals, WHO has proposed a woman- and infant-focused model of intrapartum care. The nine dimensions of this model are as follows: (1) respectful labor and childbirth care, (2) emotional support from a companion of choice, (3) effective communication by the staff, (4) pain relief strategies, (5) regular monitoring of labor, documentation, auditing, and feedback, (6) receiving oral fluids and food, (7) maternal mobility and birth position of choice, (8) a pre-established referral plan, and (9) continuity of care (12, 13). To implement this model more easily and effectively, the WHO developed and designed a next-generation partograph known as the “Labor Care Guide” (LCG) for healthcare providers to monitor the well-being of mothers and newborns during labor. This tool aims to ensure the provision of evidence-based high-quality care and stimulate shared decision-making by healthcare providers and women (15).

Studies in Iran show that there is no effective and acceptable communication between staff and pregnant women in the maternity wards (16, 17). Also, most Iranian mothers (75%) report one or more instances of non-respectful maternal care (18). Studies show that in settings with poor labor resources and a lack of multidisciplinary teamwork, implementing communication interventions during labor and childbirth may not be feasible in the long term (19, 20). According to the WHO, policy development that promotes the provision of high-quality care requires addressing multiple areas of maternity respectful care and effective staff communication with pregnant women during labor and childbirth. One of the most important measures in this direction is improving interactions at the level of the health system and providing multidisciplinary care. Also, holding regular multidisciplinary meetings to discuss and review communication approaches with women during labor and childbirth is one of the other recommendations of the WHO in this regard (13).

Maternal satisfaction with previous childbirth experiences can play a major role in a woman's decision about the type of childbirth (21). For example, dissatisfaction with the first childbirth experience may increase the desire for a cesarean section in future deliveries (18). The literature review did not yield any studies that dealt with all the components of the 2018 WHO intrapartum care model. Moreover, few qualitative studies were conducted on this subject, most of which were not comprehensive and discussed only some components of this model such as respectful maternal care and continuity of care (22–24). In line with the population policies of the Iranian government and the promotion of intrapartum care, this study on the 2018 WHO intrapartum care model is expected to lead to an increase in women's positive experiences of labor and their desire for giving birth to another child through normal vaginal delivery and also to reduce the rate of unnecessary cesarean sections.

### Study aim

Implementation and evaluation of the 2018 WHO intrapartum care model.

## Methods

### Study design

**Figure 1:**
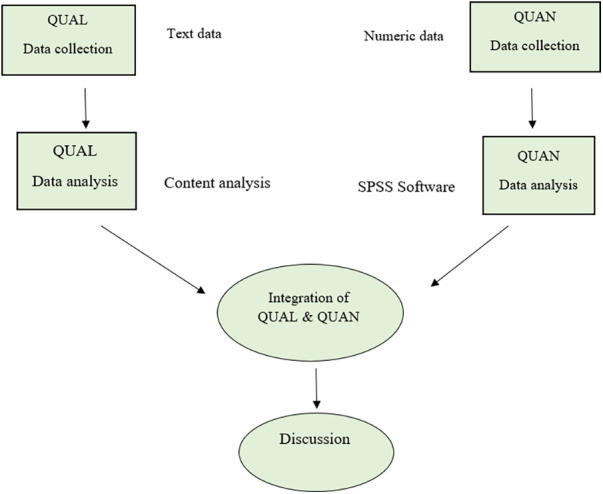
Study visual diagram.

This convergent parallel mixed-methods study will be carried out in two phases: the quantitative phase (a quasi-experimental study) and the qualitative phase. The qualitative and quantitative data will be collected and analyzed simultaneously but independently. The data relating to the two phases will be analyzed separately but the results will be combined when interpreting the data (Figure 1).

### Study phases

#### Quantitative phase

The quasi-experimental design was opted for instead of a randomized control trial because the intervention under study incorporates an accepted and well-established intervention and also, to sidestep the increased risk of contamination in the control group (25). The quantitative phase will be conducted on the pregnant women admitted to the maternity wards of Alzahra and Taleghani hospitals in Tabriz City.

#### Study settings

This study will be conducted in two public and also, specialized obstetrics and gynecology hospitals in Tabriz City, Iran (Alzahra and Taleghani). The necessary arrangements were made with the officials of the hospitals and the availability of the required equipment will be ensured before the beginning of the project. In case of any problem during the execution of the study, it will be reported. Alzahra Hospital is a Medical Education and Research Center and also, is one of the gynecological and obstetric referral centers in the northwestern region of the country. This center has 10 labor and delivery rooms (LDR). Taleghani Hospital is another Medical Education and Research Center in Tabriz and generally, mothers with low-risk conditions are admitted there. This center has eight LDRs. Each LDR in these centers is equipped with mother and neonate resuscitation facilities, fetal heart rate monitoring, a warmer, a suction device, a bathroom, and a birth ball. Also, pharmacological pain relief methods are performed at the request of mothers.

#### Specific objectives

1. To compare the childbirth experience between the intervention (receiving intrapartum care based on the WHO model) and control group (receiving routine hospital care)
2. To compare the fear of childbirth between the study groups
3. To compare the quality of intrapartum care between the study groups.

#### Secondary objectives

1. To compare the postpartum depression between the study groups
2. To compare the PTSD six weeks after childbirth between the study groups
3. To compare the desire to give birth to another child six weeks after childbirth between the study groups
4. To compare the frequency of normal vaginal delivery between the study groups
5. To compare the mean Apgar score for the infants between the study groups
6. To compare the duration of the active stage of labor between the study groups
7. To compare the duration of the second stage of labor between the study groups
8. To compare the duration of the third stage of labor between the study groups
9. To compare the exclusive breastfeeding 4–6 weeks after childbirth between the study groups
10. To determine the face, content, and construct validity and also the reliability of the Pregnancy and Childbirth Questionnaire (PCQ).

### Inclusion and exclusion criteria

The inclusion criteria are the first or second delivery of women and before the beginning of the active phase of labor (cervix dilation equal to 5 cm). The exclusion criteria are multiple pregnancies, non-cephalic presentation, indications for a cesarean section (e.g., abnormal presentation, placenta previa, etc.), obstetric problems (such as placenta previa, vaginal birth after cesarean, placental abruption, and preeclampsia), maternal underlying conditions such as cardiovascular disease and diabetes), maternal mental disabilities and other mental health problems, and the death of a loved one over the last three months.

### Sample size

The sample size was calculated in G-Power based on the two primary outcomes of the childbirth experience and fear of childbirth. According to the findings of Shakarami et al. (26) regarding the fear of childbirth and assuming *M*_1 _= 69.3, *M*_2 _= 58.9 (considering a 15% clinically significant increase caused by the intervention based on expertise panel), SD_1 _= SD_2 _= 8.5, two-sided *α* = 0.05, and test power = 95%, the calculated sample size (*n*) was 19. Moreover, according to the findings of Ghanbari et al. (27) about childbirth experience and assuming *M*_1 _= 2.71, *M*_2 _= 3.25 (considering a 20% clinically significant increase caused by the intervention based on expertise panel), SD_1_ = SD_2_ = 0.73, two-sided *α* = 0.05, and test power = 95%, the sample size (*n*) increased to 49. Finally, assuming an attrition rate of 10%, the final sample size (*n*) in each group will be 54. This sample size has the power of 99% to test between-group differences in terms of fear of childbirth.

### Sampling

To collect the data, the researcher would refer to the maternity wards of Al-Zahra and Taleghani hospitals, and the participants will be selected from among those who meet the inclusion criteria through convenience sampling. After briefing the eligible women on the research objectives and procedure, written informed consent will be obtained from those who are willing to participate in the study. The participants will be randomized to the control or intervention groups at the beginning of the active phase of labor.

### Randomization and allocation concealment

The participants will be assigned to the study groups by stratified block randomization method (first or second childbirth of woman) in blocks of 4 and 6 and with a 1:1 allocation ratio. For allocation concealment, the intervention name will be written on pieces of paper and put in a series of numbered sealed opaque envelopes. The envelopes will be kept by the head of the maternity ward. After entering the qualified woman and obtaining the informed consent, her name and medical file number will be written on the envelope by the researcher and it will be opened by the head of the ward. After determining the type of intervention, the envelope will be returned to the head of the ward.

### Blinding

It is noteworthy that the nature of this study does not allow for blinding the researcher and participants. To blind data collectors, the questionnaires for the postpartum stage will be completed by the researcher's assistant. Also, the data analyst will be blinded to group assignments.

### Intervention

After assigning the participants to the study groups, socio-demographic and obstetrics characteristics questionnaires will be filled out for all of them. Then the researcher will implement the WHO intrapartum care model for those in the intervention group, whereas those in the control group will receive routine care by staff. The implementation of the study is in the form of multidisciplinary teamwork. The researcher who will implement the intrapartum care model is PhD student of midwifery (first author). She has attended workshops on making childbirth pleasant and physiologic childbirth and also worked for four years as a physiologic childbirth tutor in a health center. In the case where advice is needed or in unpredictable high-risk cases, the gynecologist involved in the study project will be contacted (second & fourth authors). The implementation of care will be monitored by the project supervisors who are reproductive health specialists (third and corresponding authors).

The intervention based on the 9 components of the WHO intrapartum care model is as follows:

1. Respectful labor and childbirth care: To implement this component, a respectful care scale will be used. Four sub-domains of this scale included: friendly care, abuse-free care, timely care, and discrimination-free care. Calling the mother by her first name, talking to her in a familiar language, respecting her beliefs and values, not delaying the provision of care, and behaving without discrimination are the features of respectful care (28).
2. Emotional support from a companion of choice: A companion selected by the participant (in the intervention group) will be present during the labor in coordination with Metron and the supervisor. A previous study proposed that the participant's companion should take the following responsibilities: Support the woman (stay with her, calm her down, massage her, show her affection, and encourage and stimulate her), behave correctly when the woman faces fatigue, anxiety and worry, cries or screams or feels helpless, observe the regulations (wear standard clothes, avoid eating, smoking, or touching the equipment and devices), and inform the staff whenever it is necessary to leave the hospital). The companion can request information from the staff. It has been also emphasized that the companion must respect the privacy of other women (29). In this study, all these items will be recommended to the selected companion of the pregnant woman, and the researcher will monitor their observance by the companion. In cases where no one can accompany the participant, the researcher will play this role for her if she agrees. Considering that the companion's role will be to provide supportive care to the woman (as mentioned above), it seems that performing this role does not conflict with the tasks of the researcher in labor.
3. Effective communication by staff: Effective communication by staff: characteristics of effective communication according to WHO include the following: (a) informing women and their families about the advantages and disadvantages of methods and use/non-use of technologies during maternity care; (b) listening respectfully to women and their families; (c) participation women and their families in decision-making and respecting their preferences (13).
4. Pain relief strategies: The researcher in this study will employ non-pharmacological pain relief techniques, such as teaching diaphragmatic breathing with proper inhalation and exhalation, thermotherapy, position change, and massage (30) if the participant agrees. If necessary, pharmacological pain relief methods will be used at the discretion and under the supervision of a gynecologist involved in the project.
5. Regular monitoring of labor, documentation, auditing, and feedback: Both the maternity ward staff and the researcher will document the labor and childbirth events. In addition, the researcher will closely and regularly monitor obstetric care during labor, childbirth, and in the first two hours after delivery and then provide necessary feedback. Regular monitoring of labor includes measures such as intermittent control of the fetal heart rate using a fetal doppler, injection of uterotonic agents (oxytocin or misoprostol), and controlled umbilical cord traction to prevent postpartum hemorrhage, delayed umbilical cord ligation, and regular postpartum maternal monitoring for vaginal bleeding, uterine tonus, and vital signs (15). Labor documentation involves activities such as the use of partograms.
6. Receiving oral fluids and food: The participants' companions in the intervention group will be provided with a recommended list of easily digested foods or liquids to prepare (e.g., drinking water, juices, dates, biscuits, and cakes). However, the participants will be free to consume whatever they desire in small amounts divided into several portions.
7. Maternal mobility and selection of the position of choice: According to a previous study, sitting, walking, semi-sitting, four-legged, and lateral positions (both sides) will be considered for the participants in the intervention group during the first phase of labor (31). The researcher will train the participants in each of these positions and explain to them its advantages. The participants will be asked to begin with any position that is easier for them, hold each position for 10 min, and take a 10-min rest between two positions. They should also repeat these five positions at 5-cm, 7-cm, and 10-cm of cervical dilatation. Based on a previous study, the participants in the intervention group will be recommended to walk for an hour several times a day on average depending on their tolerance and duration of labor (32). Eventually, the amount of mobility and type of positioning will be at the mother's discretion
8. A pre-decided referral plan: Since the research setting includes sub-specialty hospitals, there will be no need for referral for the participants to receive higher levels of care. Nevertheless, they will be regularly monitored during labor and in the early postpartum period to coordinate with the gynecologist as soon as possible if necessary and make prompt decisions. It should be noted that considering the great importance of regular examination of women during labor and delivery, this component of the WHO model will be performed in both control and intervention groups.
9. Continuity of care: To observe the continuity of care in this study, the researcher will provide the participants of the intervention group with antepartum, intrapartum, and postpartum care one day after childbirth in the maternity ward, on the tenth day, and the fortieth day after delivery.

### Routine care

Routine care in the study settings often includes medical procedures ordered by residents of gynecology. Midwives often take care of several women and opportunities for providing midwifery-led care are very limited. Despite a clear definition of the components of respectful care, not all women receive this model of care, and other components of the intrapartum care model are not prioritized as much as medical and clinical care.

The obstetric information of the participants (e.g., duration of the active phase of labor, duration of the second and third phases of labor, Apgar score, etc.) will be also recorded during and immediately after labor. Moreover, Wijma's delivery fear scale (DFS) will be filled out for the participants before the active phase of labor and then at 7–8 cm of cervical dilation. The participants of both groups will be followed up for 6 weeks after labor and then they will be invited to a relatively quiet place to complete the Childbirth Experience Questionnaire (CEQ 2.0), the Edinburgh Postpartum Depression Scale (EPDS), the Post-Traumatic Stress Disorder (PTSD) Symptom Scale (PSS-I), the Pregnancy and Childbirth Questionnaire (PCQ), and a checklist on willingness to give birth to another child. If answering all the postpartum questions is out of the mother's patience, the interview will be conducted on two separate dates determined by the mother.

### Outcomes

Primary outcomes included birth experience, fear of childbirth, and mother's satisfaction with the quality of intrapartum care.

Secondary outcomes included postpartum depression, post-traumatic stress disorder, desire for giving birth to another child, normal vaginal childbirth, Apgar score, exclusive breastfeeding, duration of the first stage of labor, duration of the second stage of labor, duration of the third stage of labor, and face, content, and construct validity and also the reliability of the Pregnancy and Childbirth Questionnaire (PCQ).

### Data collection tools

A socio-demographic characteristics questionnaire: This questionnaire will consist of questions about the participant's age, spouse's age, age at marriage, educational attainment, job status, religion, ethnicity, marital status, housing status, household income, place of residence (rural or urban areas), weight before pregnancy, smoking, alcohol, and drug abuse before and during pregnancy.

Obstetrics history questionnaire: This questionnaire contains questions about gestational age, number of pregnancies, number of abortions, attendance at pregnancy classes, pregnancy status (wanted or unwanted), type of previous delivery (if there have been previous pregnancies), and history of pregnancy complications.

Childbirth Experience Questionnaire (CEQ2.0): This 25-item questionnaire measures four main domains of women's childbirth experience: own capacity (sense of control, personal feeling about childbirth, and labor pain), professional support (obstetric information and care), perceived safety (a sense of security and childbirth memories), and participation (a woman's ability to control her postures and movements and relieve pain during labor and delivery). Twenty-two of the 25 items in this questionnaire are scored using a 4-point Likert Scale (1: I agree, 2: I often agree, 3: I often disagree, and 4: I disagree) and the remaining 3 items are scored based on a Visual Analogue Scale (VAS) (1, 2, 3, or 4 points are given to scores 0–40, 41–60, 61–80, and 81–100, respectively). The validity and reliability of the CEQ 2.0 have been proven in a population of American women. The items with negative concepts (experiencing severe pain, fatigue, fear, and having a bad memory) receive negative scores. Higher mean scores on this questionnaire indicate a more positive childbirth experience (33).

Delivery Fear Scale (DFS): This scale was developed by Wijma to measure women's fear of childbirth during labor (34). It is a valid self-assessment questionnaire that measures fear of childbirth during labor through scores ranging from 1 (totally disagree) to 10 (totally agree). Higher scores indicate higher levels of childbirth fear (35).

Post-Traumatic Stress Disorder (PTSD) Symptom Scale (PSS-I): This scale consists of 17 items that are scored using a Likert scale. The subscales of PSS-I are re-experienced symptoms (4 items), avoidance symptoms (7 items), and symptoms of motivational reactions (6 items). If the respondent exhibits one or more re-experiencing symptoms, three or more avoidance symptoms, and two more symptoms of motivational reactions, he/she is diagnosed with PTSD. The total score on this scale ranges between 0 and 51 (36).

Edinburgh's Postpartum Depression Scale: This scale was developed by Cox et al. for measuring prenatal and postpartum depression in women. It consists of 10 four-choice questions, some of which are scored from low to high levels of depression (items 1, 2, and 4) and others are scored from high to low levels of depression (items 3, 5, 6, 7, 8, 9, and 10) with a score ranging from 0 to 3 is given to each choice, and the minimum and maximum total scores on this scale are 0 and 30, respectively. The validity of this scale was assessed by calculating the coefficient of correlation between this scale and Beck's Depression Inventory; the correlation coefficient for these tools was obtained at 0.78. In addition, the reliability of this scale was tested by calculating Cronbach's alpha and using split-half reliability; the results showed that the estimated overall reliability of this scale was 0.75 (37).

The checklist on willingness to give birth to another child: This checklist includes one Yes/No question about the women's willingness to give birth to another child.

Pregnancy and Childbirth Questionnaire (PCQ): This 25-item questionnaire was developed by Truijens et al. to measure the postpartum views of mothers about the quality of pregnancy and childbirth care. Of the 25 items of PCQ, 18 items measure experiences and perceptions of pregnant women about the quality of prenatal care in two domains: personal behavior (11 items; Cronbach's alpha = 0.87) and educational information (7 items; Cronbach's alpha = 0.90). The remaining 7 items measure puerperal women's experiences with the quality of intrapartum care (Cronbach's alpha = 0.88) and just these items will be used in this study (38). The items of this questionnaire are formulated in positive and negative propositions and scored using a 5-point Likert scale, from 1: totally agree to 5: totally disagree. The total score on PCQ ranges between 25 and 125, and higher scores indicate higher levels of satisfaction (35). The 7 items of this questionnaire that measure puerperal women's experiences of the quality of intrapartum care will be used in this study.

Implementation success rate checklist: This checklist will be designed to determine the success rate of implementation of each intervention component for each woman. This rate will be calculated by determining the answer yes in case of implementation or no in cases of non-implementation due to mothers' unwillingness or other reasons.

Partogram: A Partogram is a simple but valid chart used to monitor labor and prevent prolonged and obstructed labor focusing on observations related to maternal and fetal conditions as well as the labor progress. It provides the healthcare personnel with a pictorial overview of the labor. The information recorded on the Partogram includes maternal health, fetal health, childbirth process, and childbirth management. Partograms form an early warning system that greatly helps decision-making on the timely referral of pregnant women (39). Length of labor stages, spontaneous vaginal childbirth, the use of oxytocin, analgesia, amniotomy or episiotomy, degree of perineal tears, and also the Apgar score will be obtained from this form.

All questionnaires will be completed through interviews in a quiet place. Due to the large number of questionnaires, implementation of those will be tested prior to the study. If the interview is long and the mother is tired, two sessions will be considered.

### Validity and reliability of measurement instruments

The validity of the socio-demographic characteristics questionnaire and the obstetrics history questionnaire will be assessed through content and face validity. The literature review shows that the psychometric properties of all the measurement instruments of this study including Childbirth Experiences Questionnaire version 2.0 (CEQ 2.0) (40), Delivery Fear Scale (DFS) (41), PSS_I (PTSD Symptom Scale 1) (35), Edinburgh's Postpartum Depression Scale (42) except PCQ, have been evaluated and confirmed in Iran.

The literature review showed that the psychometric properties of the PCQ have not been assessed in Iran. To evaluate the psychometric properties of the PCQ, the necessary written permission will first be obtained from the developer of this measurement instrument. Its validity will then be assessed using translation validity (forward and backward translation) as well as content validity, face validity, and construct validity. For translation validity, the items will be first translated semantically from English to Farsi by at least two translators fluent in Farsi and English. The translated version will then be reviewed by another translator. The next step will be to translate the reviewed version from Farsi to English by one to three translators fluent in both languages who were not involved in the translation of the original version to Farsi. The final version will be then revised by three to four translators (a language specialist, an expert in questionnaire translation, a specialist familiar with concepts, and a coordinator) (43).

Face validity of the PCQ will be assessed by using a qualitative method (examining the items to identify possible ambiguities, inadequacy, and difficulties in understanding and also to ensure the appropriate fit and relevance of the items) and a quantitative method called impact score (impact scores greater than 1.5 are considered acceptable).

Moreover, a qualitative method (checking the grammar, vocabulary, importance, and placement of items in the appropriate places and also the time required for completing the questionnaire) and a quantitative method (calculating the CVR [Content Validity Ratio] and the CVI [Content Validity Index]) will be employed for evaluating the content validity of this questionnaire.

Construct validity will be examined by exploratory factor analysis (EFA) and confirmatory factor analysis (CFA). Moreover, the test-retest method will be used to assess the reliability of this tool in terms of reproducibility (ICC = intra-class correlation) and internal consistency (Cronbach's alpha). The data needed for the evaluation of research instruments will be collected 4–6 weeks after delivery.

Considering the 25 items of the PCQ, the minimum sample size for validation should be 125. Nevertheless, since the cluster sampling method will be used in this study, and considering a design effect equal to 2, the final sample size will be equal to 250 postpartum women (4–6 weeks after childbirth). After briefing the participants on the research objectives and procedure and getting their written consent, they will be asked to fill out the PCQ.

### Data analysis

The obtained data will be entered into the software using the double data entry approach and will be statistically analyzed in SPSS 24. The socio-demographic and obstetric information will be described using descriptive statistics, i.e., frequency, percentage, and mean (standard deviation) if the data follow a normal distribution pattern or median (the 25th to 75th percentile) if they do not follow a normal distribution pattern. The independent t-test and the chi-square test in the bivariate analysis and the general linear model (after controlling for the socio-demographic and obstetric characteristics) in the multivariate analysis will be also employed to compare the study groups in terms of the childbirth experience, fear of childbirth, postpartum depression, PTSD symptoms, duration of childbirth procedure, Apgar score (one and five minutes after birth), willingness to give birth to another child, and type of delivery. The missing data will be handled by the multiple imputation method). The sensitivity analysis will be done by comparing the results of the modified intention to treat analysis with the imputed data analysis.

### Qualitative phase

To explore a deeper understanding of mothers' views on the effect of implementing the study intrapartum care model, especially the components of the model that are more subjective such as respectful care or effective communication by the staff, in addition to data collection through questionnaires in the quantitative stage, interviews will also be conducted through open-ended questions with mothers of the intervention group 4–6 weeks after childbirth.

### Specific objective

Exploring the mothers' views on the effect of implementing the WHO intrapartum model on their childbirth process.

### Participants

Participants in this study will be women who had given birth (primiparous or multiparous) through the natural childbirth method with the WHO care model during childbirth and had passed 4–6 weeks postpartum, with a live and healthy infant. They will be also willing and able to describe their experiences of labor and childbirth. Purposeful sampling will be used for participant recruitment. Purposeful sampling allows the researcher to obtain rich information about a specific research question (44). Awareness of women's willingness to participate in the study will be achieved through telephone contact with the researcher. If they express interest in participating, a face-to-face meeting will be arranged at their preferred location, where the objectives and implementation process of the study, including their rights and the confidentiality of recording and storing the names and interview content, will be explained. Written informed consent will be obtained from the participants.

### Data collection

A semi-structured interview guide with open-ended questions will be used to explore and gain a better understanding of the topics and to elicit deeper insights from the participants Qualitative data will be collected through in-depth, semi-structured individual interviews with open-ended questions in an appropriate place the participants will choose. Conducting interviews with women will be done by the research assistant to avoid possible conflicts of interest. The questions to be asked are designed with the cooperation of the research team, and the ways to obtain valid data and focus on research questions are reviewed with the members of this team. The interview will begin with general questions such as, “How was your recent childbirth experience?” “Were you satisfied with the care you received during childbirth?” “To what extent did the childbirth care meet your expectations?” “What were the positive/negative factors in your childbirth experience?” Based on the type of response to each question, further probing will be done to find out the depth of the women's experiences, such as “Can you please provide more explanation and possibly give an example so that I can understand your point?”. Non-verbal data such as tone of voice, facial expressions, and body language of the participants will be also recorded by the researcher during the interview on a specific sheet, mentioning the time and location of the interview.

### Data analysis

The data obtained in this phase will be analyzed using qualitative content analysis. To this end, the researcher will read the transcript of the interviews several times to gain a general sense of the participant's experience. Then the objective words in the text that seem to convey the main thoughts or concepts will be identified for extracting the codes. After taking notes of the initial analysis and initial ideas and thoughts, the tags that reflect more than one main idea will be gradually extracted. The codes will then be classified based on their differences and similarities to form general categories.

Qualitative content analysis with a conventional approach will be used to analyze the data. Content analysis is a method for analyzing various types of data, such as visual and verbal data, and allows for the reduction of phenomena or events into defined categories to better analyze and interpret them. This method involves a deep understanding of the content through careful reading of sentences multiple times, identifying codes, searching for sub-themes, reviewing, and ultimately interpreting themes (45). The phrases and sentences related to the mothers' childbirth experiences will be coded on the margins of the coding sheets. Coding will be primarily based on the text using the mothers' descriptions. Then, codes with similar content will be gathered into sub-themes and themes. The authors will discuss and examine the interpretations of the mothers' descriptions of their childbirth experiences and reach an agreement on the themes.

### Trustworthiness of the findings

To increase the trustworthiness of the data, four criteria of dependability, credibility, transferability, and confirmability will be considered in conducting the research (46). To ensure the dependability of the data, maximum diversity in terms of age, education, occupational status, and socio-economic status will be considered in selecting participants. Additionally, the member check technique, also known as participant validation, will be utilized. For this purpose, the data or findings will be returned to the participants to validate them against their own experiences. Furthermore, the data will be made available to the research team for comparison and to ensure alignment of themes with participants' statements, and their opinions regarding codes, themes, and analyses will be collected in written form. To facilitate access to credible data and create a sense of trust and comfort among the participants during the interviews, conditions will be provided for them to express their opinions openly and honestly. To increase transferability and allow judgments about the fit of the research context with other fields, detailed descriptive information about the method and background will be included in the research report. Additionally, by considering the various age, education level, and occupations of the participants, the transferability of the study findings will be enhanced. To increase confirmability, all stages of the research will be documented, enabling other researchers to follow up on the data.

### Data safety and monitoring board (DSMB)

Because this is a clinical trial that does not involve a medicinal product, a DSMB is not required.

### Ethical considerations

This study has been approved by the ethics committee of Tabriz University of Medical Sciences (ethics code: IR.TBZMED.REC.1401.093). The quantitative phase of the study (a quasi-experimental study) has been registered in the Iranian Clinical Trial Registration Centre (IRCT): IRCT20120718010324N69. URL: https://fa.irct.ir/user/trial/68313/view. All participants will be given verbal consent for participation in the study after the consent form is read to them. Written informed consent will be obtained from all participants in a face-to-face session following the Declaration of Helsinki. When the participant is illiterate, it will be signed by a literate witness who makes sure that it is understood and confirmed that consent will be given freely (47). They will be assured of the confidentiality of their information and privacy. It will also be explained that they are allowed to drop out of the study at any stage of the intervention and that refusing to cooperate at any stage is free and that there will be no change in the provision or the quality of services provided to them.

## Discussion

Well-developed and evidence-based policies are essential to ensure safety and quality of care (48). Most policies on maternal care emphasize that all women and their infants should receive evidence-based, equitable, compassionate, and respectful care during childbirth. Nonetheless, the reality is sometimes different, and all mothers and infants, both in rich and poor countries, do not have positive experiences of childbirth. Moreover, it is not possible to make fundamental interventions worldwide (12).

In line with the third goal of sustainable development, there is a global shift in maternal and neonatal health programs from focusing on survival to realizing care programs. High-quality clinical care, improved communication, education, information, and respect from healthcare providers are essential aspects of pregnant women's care. A combination of these factors can ensure the safety of mothers and their infants (49). This study will probably face some challenges. The effective implementation of this model requires the active participation of women and the cooperation of the maternity ward with the researchers. Also, the occurrence of unpredictable high-risk conditions during the labor and childbirth process may cause problems in the implementation of the intervention component. Given that there are only two maternity centers affiliated with the University of Medical Sciences in Tabriz City, this study will be conducted in only two centers. Due to the nature of the intervention, it will not be possible to blind the participants and researcher.

### Study implication

Considering the population policies pursued by the Iranian government that require an increase in childbearing, it is necessary to adopt programs to improve the quality of services provided to pregnant mothers. Maternal satisfaction with childbirth can be measured by midwifery intrapartum care (9). If positive and effective results are obtained from this pilot study, this care model can be implemented on a wider level using the WHO Labor Care Guide after holding training courses for staff. The WHO model of intrapartum care can encourage the development or revision of intrapartum care programs for expectant mothers to protect the human dignity and rights of mothers, increase their positive experiences of childbirth, and motivate them to give birth to another child. Additionally, the high rate of unnecessary cesarean sections is among the main factors bringing down the values of health indicators. As a result, the implementation of such care models is expected to increase women's desire for normal vaginal childbirth, prevention of mental disorders caused by negative experiences of childbirth, and also, prevent uncontrolled increases in cesarean sections.

### Access to data and results

The research group will convey the results to participants, healthcare and public health professionals, and other relevant groups through publication.

## Ethics statement

This study has been approved by the ethics committee of Tabriz University of Medical Sciences (ethics code: IR.TBZMED.REC.1401.093). The quantitative phase of the study (a quasi-experimental study) has been registered in the Iranian Clinical Trial Registration Centre (IRCT): IRCT20120718010324N69. URL: https://fa.irct.ir/user/trial/68313/view.
